# What are the differences among occupational groups related to their palliative care-specific educational needs and intensity of interprofessional collaboration in long-term care homes?

**DOI:** 10.1186/s12904-017-0207-y

**Published:** 2017-05-18

**Authors:** S. Kaasalainen, T. Sussman, M. Bui, N. Akhtar-Danesh, R. D. Laporte, L. McCleary, A. Wickson Griffiths, K. Brazil, D. Parker, V. Dal Bello-Haas, A. Papaioannou, J. O’Leary, the SPA-LTC Team

**Affiliations:** 1 Faculty of Health Sciences, 3N25F, McMaster University, 1280 Main Street West, Hamilton, ON L8N 3Z5 Canada; 2 McGill University, Montreal, QC, Canada; 3 University of Toronto, Toronto, Canada; 4 Brock University, St. Catharines, ON Canada; 5 University of Regina, Regina, SK Canada; 6 Queen’s University Belfast, Belfast, UK; 7 Deborah Parker, University of Western Sydney, Sydney, Australia

**Keywords:** Palliative care, Long-term care, Interprofessional care, Aged care

## Abstract

**Background:**

The purpose of this study was to compare the differences across occupational groups related to their end-of-life care-specific educational needs and reported intensity of interprofessional collaboration in long-term care (LTC) homes.

**Methods:**

A cross-sectional survey, based on two questionnaires, was administered at four LTC homes in Ontario, Canada using a modified Dilman’s approach. The first questionnaire, End of Life Professional Caregiver Survey, included three domains: patients and family-centered communication, cultural and ethical values, effective care delivery. The Intensity of Interprofessional Collaboration Scale included two subscales: care sharing activities, and interprofessional coordination. In total, 697 LTC staff were given surveys, including personal support workers, support staff (housekeeping, kitchen, recreation, laundry, dietician aids, office staff), and registered staff (licensed nurses, physiotherapists, social workers, pharmacists, physicians).

**Results:**

A total of 317 participants completed the survey (126 personal support workers, 109 support staff, 82 registered staff) for a response rate of 45%. Significant differences emerged among occupational groups across all scales and subscales. Specifically, support staff rated their comfort of working with dying patients significantly lower than both nurses and PSWs. Support staff also reported significantly lower ratings of care sharing activities and interprofessional coordination compared to both registered staff and personal support workers.

**Conclusions:**

These study findings suggest there are differing educational needs and sense of interprofessional collaboration among LTC staff, specific to discipline group. Both the personal support workers and support staff groups appeared to have higher needs for education; support staff also reported higher needs related to integration on the interdisciplinary team. Efforts to build capacity within support staff related to working with dying residents and their families are needed. Optimal palliative care may require resources to increase the availability of support for all staff involved in the care of patients.

## Background

As a unique health care environment with medically complex older adult residents, significant challenges exist in establishing a national end of life strategy for Canadian long-term care (LTC) homes that is integrated with non-LTC palliative care services [1–4]. Twenty-seven percent of Canadian residents will die in LTC annually [5] and this rate is expected to increase to 39% by 2020 [6]. Currently, Canadian LTC homes have insufficient resources to meet the needs of their dying residents with 19.1% of LTC residents dying in acute care and 40.7% being hospitalized within 6 months prior to death [7]. National LTC staff-to-resident ratios remain significantly lower (5 h per resident per day) than other palliative care delivering facilities, with Ontario ranking consistently below national averages (4 h per resident per day) [8].

Most LTC residents die from non-cancer conditions, such as co-occurring dementia, heart failure, and chronic obstructive pulmonary disease, which have not traditionally been major focuses of study in palliative care research [9–12]. Cognitive, communication, functional, and behavioural barriers to delivering effective palliative care exist in LTC since over 75% of residents have some degree of cognitive impairment [13–16]. Pain and other symptoms are often poorly managed in LTC [17], which is especially evident among residents with advanced dementia. Furthermore, LTC residents are among the frailest and most vulnerable older adult populations with approximately 52.3% (95% confidence interval 37.9%–66.5%) of LTC residents being classified as frail and 40.2% (28.9%–52.1%) being considered pre-frail [18, 19]. As a result, many palliative care tools and approaches primarily developed from cancer care research have limited applicability in LTC settings.

An interprofessional collaborative approach has been supported and strongly encouraged by health care workers [3], law and policy makers [7, 20] and researchers [10, 17, 21–24] as an essential component for addressing the complex physical, psychosocial, emotional and spiritual needs of LTC residents undergoing palliative care. The Canadian Interprofessional Health Collaborative (CIHC) defines *interprofessional collaboration* as the “process of developing and maintaining interprofessional working relationships with learners, practitioners, patients/clients/families and communities to enable optimal health outcomes” [23]. Evidence on the interprofessional collaboration, however, is especially sparse in the context of LTC and requires further study to establish the effectiveness of practice-based interventions.

Unlike other health care settings, physician involvement is usually very minimal in LTC and other regulated health professionals, such as pharmacists, dieticians, physiotherapists, and occupational therapists, are not regularly onsite [1]. LTC staff primarily consists of unregulated health care workers with limited training and education including personal support workers, dietary aides, recreational aides, and chaplains [1]. These unregulated health care workers are rarely examined or considered in studies regarding interprofessional palliative care in LTC settings. The lack of regulation amongst many of the core team members and low ratios of regulated health professionals create challenges in developing, reinforcing, and evaluating the therapeutic quality of interprofessional palliative care programs in LTC. Thus, in order to improve staff capacity to communicate with families and residents about end of life issues and deliver effective palliative care services, it is imperative to know how comfortable different LTC workers are regarding palliative care delivery and the nature of LTC as a unique collaborative environment.

The **aim of this study** was to compare the differences across occupational groups related to their palliative care-specific educational needs and intensity of interprofessional collaboration in long-term care (LTC) homes. Study data and findings reported in this paper are part of a larger mixed methods study that is currently exploring the implementation of a palliative program, called Strengthening a Palliative Approach in Long Term Care (SPA-LTC). This paper reports on the analysis of survey data that was collected at baseline from the four participating LTC homes in the SPA-LTC program.

## Methods

### Design

A cross-sectional survey design was used to examine the educational needs and intensity of interprofessional collaboration among LTC staff. This study was approved by three university-affiliated Research Ethics Boards in two provinces of Canada.

### Setting and sample

Data were collected from staff at four LTC homes in southern Ontario in 2015. The facilities were purposively chosen to represent a set of diverse conditions in LTC (e.g., for-profit/not-for profit status, facility size). Staff were grouped into the following categories: Personal Support Workers (PSWs) or nursing care aides; Support Staff (i.e., housekeeping, kitchen, cooks, recreation, laundry, dietician aid, office/administrative staff (who are not registered staff), reception); Registered Staff (i.e., licensed nurses, physiotherapists, social workers, dieticians, pharmacists, physicians).

### Measurement

The survey included two questionnaires. The *End-of-life Professional Caregiver (ELPC)* survey and the *Intensity of Inter-Professional Collaboration (IPC)*. The ELPC was developed to assess palliative care-specific educational needs within an interprofessional team related to: (a) clinical knowledge/technical skills; (b) communication/interpersonal skills with patients, family, and other clinicians; (c) spiritual and cultural issues; (d) ethical, professional, and legal principles; (e) organizational skills; and, (f) attitudes, values and feelings of health care professionals. The ELPC is a 28-item scale with strong internal consistency (alpha = .96) [25]. Each item scored on a 5-point Likert scale ranging from 1 (lowest level of skill) to 5 (greatest level of skill). It includes three subscales: a 12-item Patient-and Family-Centered Communication (PFCC); 8-item Cultural and Ethical Values (CEV); and 8-item Effective Care Delivery (ECD) [25]. The PFCC subscale measures includes items focused on the comfort with discussing palliative issues (e.g., helping family accept a prognosis or manage conflict, goal setting, advance care planning, grieving etc) with family and/or health care professionals. Items included in the CEV subscale are focused on providing culturally and ethically competent care while ECD items include related to clinical competence (e.g., referring to hospice, familiarity with PC principles, linking with appropriate services when needed and navigating the system) and perceived workplace supports available to them to deal with palliative issues.

IP collaboration was measured using the IPC which is an 18-item scale that measures two factors: care sharing activities and IP co-ordination [26]. Initial factor analysis and validation of this scale reported that the main factors associated with interdisciplinary collaboration are most closely aligned to intragroup dynamics and values, as opposed contextual factors, such as the size of an employing program’s workforce, or whether a program formally assesses the quality of its care [26]. The survey took approximately 10–15 min to complete. Demographic and employment data was also collected, such as age, gender, length of time working in LTC, occupational group, and involvement in care planning activities.

### Procedure

We worked with the LTC administrative staff to distribute the survey via inter-facility mail to all LTC staff. We also distributed surveys at staff educational events to improve the response rate. We tracked those staff who completed the survey and followed up with those who did not with a subsequent mailing distribution. To encourage completion, we held a draw at each of the participating LTC homes and told staff that they would be entered to win a $50 gift card if they completed a survey. All completed surveys were returned to the principal investigators of the study (SK & TS).

### Statistical analysis

All statistical analysis was performed in SPSS 23.0 statistical analysis software for Windows (IBM Corp., Armonk, NY, USA). A frequency distribution was completed on demographic variables and employment responsibilities of interest (attending care conferences, contributing to the development of care plans). Individual descriptive statistics were also reported for each of the three occupational groups studied (PSW, Registered Staff, Support Staff). Mean responses were generated for each scale and their subscales according to occupational group and a stepwise regression analysis was performed to evaluate the contribution of the independent variables to these mean responses. Criteria for inclusion in the predictive model was a *P* value of <0.05. Significant predictors from the regression models were selected for between groups comparisons on survey subscale responses using ANOVA and Tukey post hoc analyses.

## Results

### Characteristics of the sample

Of the 697 surveys distributed, 317 were completed and returned to study investigators, for a total response rate of 45% (see Table 1). Response rates for the different occupational groups were 45% for the PSWs (126/317), 50% for support staff (109/219), and 55% for registered staff (82/148).

**Table 1:** Demographic and employment characteristics by occupational group

Demographic characteristics	PSWs *n* = 126	Registered staff *n* = 82	Support staff *n* = 109	^a^Total *N* = 317
Sex N (%)				
Male	1 (0.8)	14 (17.1)	26 (24.3)	41 (13.1)
Female	124 (99.2)	68 (82.9)	81 (75.7)	273 (86.9)
Age N (%)				
Under 25	1 (0.8)	1 (1.4)	3 (2.8)	5 (1.6)
25 to 34	13 (10.7)	17 (20.1)	20 (18.5)	50 (16.1)
35 to 44	28 (23.0)	26 (31.7)	28 (25.9)	82 (26.4)
45 to 54	41 (33.6)	24 (29.3)	34 (31.5)	99 (31.8)
55 to 64	34 (27.9)	10 (12.2)	19 (17.6)	63 (20.3)
65+	4 (3.3)	3 (4.2)	4 (3.7)	11 (3.5)
Highest level education completed N (%)				
High school or equivalent	35 (28.0)	2 (2.9)	26 (24.5)	63 (20.3)
College	64 (51.2)	35 (42.7)	44 (41.5)	143 (46.0)
Undergraduate degree	8 (6.4)	13 (15.9)	21 (19.8)	42 (13.5)
Graduate degree	13 (10.4)	30 (36.6)	11 (10.4)	54 (17.4)
Other	5 (4.0)	0 (0.0)	4 (3.8)	9 (2.9)
Employment status N (%)				
Full-time	69 (55.6)	54 (65.9)	76 (69.7)	199 (63.6)
Part-time	55 (44.4)	26 (31.7)	33 (30.3)	112 (35.8)
Years working in LTC mean (SD)	12.1 (9.2)	8.8 (7.9)	10.2 (7.6)	10.6 (8.5)
Years w/ current employer mean (SD)	10.6 (8.5)	5.8 (5.9)	8.2 (7.0)	8.5 (7.6)
Attended care conferences N (%)	66 (61.1)	61 (74.4)	30 (30.6)	157 (55.9)
Contributed to care plans N (%)	95 (84.8)	75 (91.5)	37 (38.5)	207 (72.1)

^a^Total number may not equal 100% due to missing responses

Staff were primarily female (86.9%) with the majority (82%) aged 35 and older. Most participants earned a college diploma or higher (79.7%) and were employed on a full-time basis (64%). The participants had a mean of 10.6 (SD = 8.5) years of experience working in LTC and a mean of 8.5 years (SD = 7.6) working with their current employer.

Fifty-six percent of participants reported that they had attended care conferences; highest among registered staff (74%) and lowest among support staff (31%). Seventy-two percent of participants reported that they had contributed to the development of care plans for residents; these rates are highest among registered staff (91%) and lowest among support staff (39%).

### ELPC and IIPC survey

Stepwise regression analysis of the ELPC subscales found that both occupation and level of education significantly predicted responses to items in the PFCC (Patient-and Family-Centered Communication) and ECD (Cultural and Ethical Values) subscales, whereas only occupation predicted response on the CEV subscale (Table 2). Stepwise regression analysis for the IIPC scale retained occupation and years spent working in LTC as significant predictors of responses on the Care Sharing Activities subscale, whereas only occupation was retained in the regression model for the Inter-Professional Coordination subscale. Interestingly, for this subscale the regression coefficient for years spent working in LTC was negative (*β* = −.012, *p* = 0.027, CI 95% [−0.022, −0.001]), suggesting that the longer staff worked in LTC, the lower their appraisal of care sharing activities across occupational groups.

**Table 2:** Stepwise regression results for mean ELPC and IIPC subscales

Subscale	β	CI 95%	*P*-value
ELPC			
PFCC			
Occupation	0.31	0.185; 0.443	0.001
Education	0.16	.054; 0.260	0.003
CEV			
Occupation	0.31	0.174; 0.448	0.001
ECD			
Occupation	0.38	0.255; 0.509	0.001
IIPC			
IP Caring			
Occupation	0.12	0.020; 0.225	0.019
Yrs in LTC	-0.01	−0.022; −0.001	0.027
IP Coord			
Occupation	0.15	0.053; 0.255	0.003

*ELPC* End of Life Professional Caregiver Survey consists of: *PFCC* Patient and Family Centered Communication; *CEV* Cultural and Ethical Values, *ECD* Effective Care Delivery, *IIPC* Intensity of Inter-professional Collaboration

*P*<0.05 is significant

ANOVAs were performed to evaluate the relationship between significant predictors in the regression models and the subscale responses. Analysis showed a significant relationship between occupational group and all three subscales of the ELPC, as well as the Interdisciplinary Coordination subscale of the IIPC. (Table 3). Subsequent Tukey post hoc tests reported significant differences between all occupational groups in the ELPC subscales (*p* < .01). Analysis of occupational groups also revealed significant groups differences in the Inter-professional coordination subscale of the IIPC (see Table 4). Subsequent Tukey post hoc tests revealed significant difference between the Support Staff and both PSWs (*p =* 0.004) and Registered Staff (*p* = 0.001). The PFCC and ECD subscales of the ELPC were compared based on different education levels. Only PFCC responses were significantly related to education level (*p* = 0.002). The relationship between education and ECD responses approached significance with a reported *p*-value of 0.053. Post hoc Tukey tests reported differences between individuals with a high school level of education compared to either college or graduate degrees. There was no difference in PFCC between the high school graduates and those who completed university-level education. No significant differences were found in the post hoc comparisons of education level on the ECD subscale responses.

**Table 3:** Analysis of variance (ANOVA) results for mean ELPC and IIPC subscale scores by occupational group

	F-score	df	SS	Significance
ELCS				
PFCC	49.20	2	76.60	*0.000*
CEV	29.86	2	55.94	*0.000*
ECD	43.85	2	69.30	*0.000*
TOTAL	47.59	2	65.99	*0.000*
IPC				
IP caring	7.02	2	7.99	*0.001*
IP coord	7.63	2	8.39	*0.001*
TOTAL	8.78	2	8.70	*0.000*

*ELPC* End of Life Professional Caregiver Survey consists of: *PFCC* Patient and Family Centered Communication, *CEV* Cultural and Ethical Values, *ECD* Effective Care Delivery, *IPC* Intensity of Inter-professional Collaboration

*P*<0.05 is significant

**Table 4:** Differences in the End of Life Professional Caregiver (ELPC) and intensity of Inter-Professional Collaboration (IPC) surveys amoung occupational groups

Survey	Support staff mean (SD)	PSW mean (SD)	Registered staff mean (SD)	All groups mean (SD)	Comparison between occupational groups		Mean difference (A-B)	*P* value
					A	B		
ELPC^a^								
					SS	RS	-1.28	*0.001*
PFCC	2.00 (1.1)	2.64 (0.8)	3.29 (0.6)	2.60 (1.0)		PSWs	−0.64	*0.049*
					PSWs	RS	−0.18	0.228
					SS	RS	-0.109	*<0.001*
CEV	1.93 (1.2)	2.59 (0.9)	3.02 (0.7)	2.48 (1.0)		PSWs	−0.66	*<0.001*
					PSWs	RS	−0.43	*0.006*
					SS	RS	-1.17	*<0.001*
ECD	1.55 (1.1)	2.35 (0.9)	2.72 (0.7)	2.18 (1.0)		PSWs	−0.80	*<0.001*
					PSWs	RS	−0.36	*0.011*
					SS	RS	-1.18	*<0.001*
Total	1.87 (1.0)	2.54 (0.8)	3.04 (0.6)	2.45 (0.9)		PSWs	−0.67	*<0.001*
					PSWs	RS	−0.51	*<0.001*
IIPC^a^								
					SS	RS	-0.42	*0.001*
IPC Caring	3.73 (0.8)	3.97 (0.8)	4.15 (0.6)	3.94 (0.8)		PSWs	−0.24	*0.049*
					PSWs	RS	−0.18	0.228
					SS	RS	-0.39	*0.001*
IPC coordination	3.73 (0.8)	4.05 (0.8)	4.12 (0.6)	3.96 (0.8)		PSWs	−0.32	*0.004*
					PSWs	RS	−0.07	0.788
					SS	RS	-0.42	<0.001
Total	3.72 (0.8)	4.00 (0.7)	4.13 (0.6)	3.94 (0.7)		PSWs	−0.29	*0.007*
					PSWs	RS	−0.13	0.405

^a^Higher scores reflect greater skill, with 5 reflecting the greatest and 1 reflecting the least

*ELPC* End of Life Professional Caregiver Survey consists of: *PFCC* Patient and Family Centered Communication, *CEV* Cultural and Ethical Values, *ECD* Effective Care Delivery, *IPC* Intensity of Inter-professional Collaboration, *SS* Support Staff, *PSW* Personal Support Workers, *RS* Registered Staff

*P*<0.05 is significant

## Discussion

These survey findings contribute to our understanding of the needs, gaps, and perspectives of LTC staff to support an interdisciplinary approach to palliative care. To the best of our knowledge, this is the first study to explore this topic with a group of licensed staff, personal support workers, and support staff.

The finding that support staff rated their comfort of working with dying patients significantly lower than both nurses and PSWs was somewhat surprising. Swinney et al. found similar results in a pediatric palliative setting; whereby support staff reported feeling uncomfortable with interactions with dying children and their families, largely due to their insufficient knowledge and training in palliative care [27]. Moreover, support staff reported that experiencing a child’s death adversely affected their lives outside of work, with 43.1% experiencing greater problems with depression since they started working with dying children, and 25% of them reporting that the death of a child had had an adverse effect on their ability to work. While it is true that support staff spend less time in care planning, attending care conferences (supported by the results of this study), they still spend a great deal of time interacting with residents and family members. For example, maintenance workers are needed to replace lightbulbs and housekeeping clean resident rooms; these activities often involve conversations with residents and/or their family members. Perhaps having these conversations without being involved in other care-related discussions that involve the typical ‘care team’, makes them feel less empowered and hence, more vulnerable, to distressing emotional responses in response to death and dying situations. Given that support staff spend 60% of their time interacting with patients and families, Swinney et al. state that organizations need to allocate resources for support staff to participate in palliative care training programs to improve their knowledge, confidence while equipping them with coping skills to deal with difficult dying situations [27].

Based on our study findings, one could argue that the caring component of support staff’s work is invisible, and hence their grief is not acknowledged by the health care team, the LTC organization or society itself. Doka coined this term ‘disenfranchised grief’, such that the relationship of support staff with LTC residents is not recognized and subsequent loss is not acknowledged, and they are excluded from ‘the grieving circle’ [28]. Spidell et al. found that 21% of chaplains felt that their grief was not supported or affirmed in the workplace [29]. Moreover, Anderson and Gaugler reported that certified nursing assistants, or personal support workers, felt excluded from grieving the loss of their patients despite the depth of their relationship with the LTC resident [30]. However, our findings suggest that personal support workers felt more supported than support staff, consistent with the proposition that disenfranchised grief is not binary (e.g., present or absent) but rather a hierarchical based on social norms about the legitimacy of bereavement based on relationships [31]. Interestingly, Wlodarczyl found that a group music intervention with hospice workers has the potential to improve grief resolution associated with disenfranchised grief [32]. Clearly, interdisciplinary palliative training programs along with other interventions aimed at resolving grief in LTC homes for support staff are needed, based on our study findings.

Interdisciplinary palliative training programs have been shown to improve collaboration in LTC [33]. In an evaluation of the Gold Standards Framework in Care Homes (GSFCH), Badger et al. found that staff reported improved knowledge of palliative care, confidence, communication and collaboration. They state that the GSFCH helped to address limitations to collaborative working, including some perceptions of unequal status and lack of trust between practitioners by providing training, networking and support. However, it is unclear whether this training was inclusive of all team members in LTC. Most commonly, teams include professional staff, such as nurses, physicians, and occasionally nonregulated staff (i.e., personal support workers) but including support staff is rare.

Interdisciplinary palliative care training programs can be delivered in a variety of ways. Wagner et al. suggest the use of interdisciplinary ‘huddles’ enable teams to have short but frequent briefings, offering a mechanism for immediate learning in LTC homes [34]. Evidence on the use of huddles in acute care shows that workplace culture, communication, collaboration and staff satisfaction improves [35]. Comfort Care Rounds, as a more formal type of ‘huddle’, have been used to provide a LTC home-wide forum for case-based discussions about deceased residents or those who are dying [36]. Pilot evaluation of Comfort Care Rounds showed that staff reported: (a) new learning about palliative care; (b) improved communication and relationships between staff members; (c) increased confidence in providing palliative and end-of-life care; (d) empowered PSWs in providing and discussing palliative care; (e) provided opportunities for debriefing and reflection; and, (f) increased awareness and use of palliative care human resources [36].

Another strategy to enhance interdisciplinary training is the use of ‘palliative champion’ teams ([37–39], http://www.palliativealliance.ca). However, to be a strong team, palliative champion team members need to have a common ideal and understanding of the contribution of that each team member makes to achieve successful team outcomes [40]. Wittenberg-Lyles found that communication in palliative team meetings tends to emphasize biomedical information sharing [41]. To offset this, team meetings should include strategic use of questions or structured guides to elicit engagement from all team members to improve interdisciplinarity, team identity, collegial decisions, and professional identity [42, 43].

Including support staff as members of the palliative champion team or as part of team huddles or palliative care program training, may facilitate improved palliative care knowledge, support and collaboration for all occupational groups who work in LTC. Efforts are beginning to focus on empowering personal support workers or care aides within a palliative approach to care [44], but these survey findings highlight the need to support other groups of staff as well, especially support workers. Although support workers may not spend as much time at the bedside as personal support workers, they interact with residents and families often and need to be supported so that they can work within a palliative approach if the need arises.

There are some limitations to this study. The results may not be generalizable to all LTC settings due to the use of convenience sampling that included only four LTC homes that were mostly in urban southern Ontario. Moreover, we were not able to capture the perspectives of physicians in these LTC homes due to their nonresponse to the survey. Future studies should use larger sample sizes over a larger geographical area. Moreover, the limitations of survey designs should be acknowledged, in particular the superficial nature of the data that is elicited. The use of rigorous qualitative methods that employ more in-depth data collection and analysis strategies would provide richer data related to LTC staff perceptions of educational and supportive needs in providing palliative care.

## Conclusions

These study findings suggest there are differing needs of LTC staff, specific to occupational group. There appears to be an implicit hierarchical nature among staff which can contribute to more disenfranchised grief, particularly for support staff. Given the nature of relationships that can be developed in LTC, more attention needs to be given to acknowledging these relationships within a supportive environment to help support staff manage their own grief and bereavement. In doing so, staff will be in a better position to support LTC residents and their family members more effectively.

## Abbreviations

LTC: long term care
PSW: personal support workers
ELPC: End of Life Professional Caregiver Survey
PFCC: Patients and Family-Centered Communication
CEV: Cultural and Ethical Values
ECD: Effective Care Delivery
IPC: Intensity of Interprofessional Collaboration Scale
IP Caring: Interprofessional Care Sharing Activities
IP Coordination: Inter-Professional Coordination
